# The dual role of autophagy in periprosthetic osteolysis

**DOI:** 10.3389/fcell.2023.1123753

**Published:** 2023-03-24

**Authors:** Zhaoyang Yin, Ge Gong, Xiang Wang, Wei Liu, Bin Wang, Jian Yin

**Affiliations:** ^1^ Department of Orthopedics, The First People’s Hospital of Lianyungang, The Affiliated Lianyungang Hospital of Xuzhou Medical University, Lianyungang, China; ^2^ Department of Geriatrics, Jinling Hospital, Medical School of Nanjing University, Nanjing, China; ^3^ Department of Orthopedics, The Affiliated Jiangning Hospital with Nanjing Medical University, Nanjing, China

**Keywords:** periprosthetic osteolysis, autophagy, mitophagy, aseptic loosening, RANKL

## Abstract

Periprosthetic osteolysis (PPO) induced by wear particles is an important cause of aseptic loosening after artificial joint replacement, among which the imbalance of osteogenesis and osteoclastic processes occupies a central position. The cells involved in PPO mainly include osteoclasts (macrophages), osteoblasts, osteocytes, and fibroblasts. RANKL/RANK/OGP axis is a typical way for osteolysis. Autophagy, a mode of regulatory cell death and maintenance of cellular homeostasis, has a dual role in PPO. Although autophagy is activated in various periprosthetic cells and regulates the release of inflammatory cytokines, osteoclast activation, and osteoblast differentiation, its beneficial or detrimental role remains controversy. In particular, differences in the temporal control and intensity of autophagy may have different effects. This article focuses on the role of autophagy in PPO, and expects the regulation of autophagy to become a powerful target for clinical treatment of PPO.

## 1 Introduction

Clinically, joint replacement is an effective procedure for the treatment of joint trauma and end-stage joint diseases, such as osteoarthritis and rheumatoid arthritis (Price et al., 2018). It is estimated that by 2030, more than 4 million joint replacement surgeries will be performed annually in the United States (Charnley, 1971; Kurtz et al., 2007). With the continuous improvement of surgical methods and the further advancement of prosthetic materials, the quality of life of patients has been greatly improved (Shan et al., 2014). Nevertheless, there are still a certain number of patients who require more difficult revision surgery, the main causes of which are periprosthetic osteolysis (PPO) and aseptic loosening (AL) (Malchau et al., 1993; Learmonth et al., 2007). Artificial joint revision surgery is also called re-joint replacement. Generally, when the joint prosthesis becomes loose or damaged, or pain occurs and affects normal life, the orthopedic doctor will open the prosthesis of the original joint and then take it out, and then treat the soft tissue and bone appropriately according to the specific situation. Finally, a suitable new joint prosthesis is placed in the joint again. In the United States, revision surgery after joint replacement surgery due to PPO accounts for 20.3% and is one of the most common reasons (Desai and Bancroft, 2008; Yin et al., 2022). In a 10-year follow-up study, osteolysis occurred in 24% (32 of 133 hips) of patients with primary total hip replacement using standard polyethylene (Lachiewicz and Soileau, 2016). In a serial follow-up of 250 single-center ankle arthroplasties, the prevalence of osteolysis was quite high at 31.6% (79 of 250 ankles) and occurred within 3 years of surgery (Lee and Lee, 2022). These results collectively indicate that PPO after joint replacement surgery is a challenging problem. PPO arises from a chronic inflammatory response triggered by cellular phagocytosis of implant-derived particulate debris. Multiple cytokines lead to osteoclast recruitment and activation, bone resorption, and ultimately AL (Looney et al., 2006; Noordin and Masri, 2012). The pathophysiology of AL has not been fully elucidated. The etiology may be related to the material and design of the prosthesis, the procedure, and the way the prosthesis is used. At present, the common prosthesis materials include metal, polyethylene, polymethyl methacrylate and ceramics (Bozic et al., 2009; Bouhidel et al., 2015). Currently, titanium (Ti)-based composites are the most ideal prosthetic implants due to their excellent mechanical strength and good biocompatibility. However, Ti implants have some inherent drawbacks and are prone to the formation of wear debris due to their high coefficient of friction (Kang et al., 2017; Bressan et al., 2019). Titanium wear particles may cause damage to surrounding cells and exacerbate the spread of inflammation. How to alleviate the death and inflammatory response of periprosthetic cells is a key way to prevent PPO.

Periprosthetic membrane (PM) refers to the synovial-like interface membrane between the prosthesis and the surrounding bone tissue, which contains a variety of cell types, such as fibroblasts, macrophages, osteoclasts, osteoblasts, osteocytes and a small number of lymphocytes (Tuan et al., 2008; Camuzard et al., 2019). Wear particles from prosthetic materials are phagocytosed by cells within the PM and induce an inflammatory response, leading to an imbalance of osteoblasts and osteoclasts, which subsequently leads to osteolysis and AL (Catelas et al., 1999; Nich et al., 2013). The cell types and molecular biological mechanisms involved are very complex, including cytokines such as interleukin 6 (IL-6), tumor necrosis factor alpha (TNF-α), interferon (IFN)-β, interleukin 1β (IL-1β), prostaglandin E2 and macrophage-colony stimulating factor (M-CSF) production, cell-to-cell interactions and ultimately the recruitment and activation of osteoclasts (Okafor et al., 2006; Wang et al., 2017; Yang et al., 2016; Deng et al., 2017a; O'Neill et al., 2013; Purdue et al., 2007; Lochner et al., 2011; Ping et al., 2017). In this series of biological activities, the release of receptor activator of nuclear kappa-B ligand (RANKL) and its binding to receptor RANK are central events, which directly promote osteoclastogenesis and subsequent osteolysis (Zhai et al., 2014; Zhao et al., 2016; Ping et al., 2017). After being stimulated by wear particles, cells in PM can undergo multiple modes of regulated cell death (RCD), including autophagy (Chen et al., 2021), apoptosis (Deng et al., 2017b), pyroptosis (Zhang et al., 2022) and even ferroptosis (Xu et al., 2021). Among them, autophagy is a well-studied RCD mode, which plays a pivotal role in PPO.

Autophagy is a ubiquitous and highly conserved process of self-catabolism and energy dynamic cycle unique to eukaryotic cells. During the process of cell proliferation, differentiation and maturation. It maintains intracellular homeostasis and provides material as well as energy by degrading misfolded proteins or damaged organelles (Glick et al., 2010; Mizushima and Komatsu, 2011). Three types of autophagy have been described in mammals, namely,: macroautophagy, chaperone-mediated autophagy and microautophagy, depending on the mechanism by which cargo is delivered to the lysosome (Scrivo et al., 2018). The current research focuses on macroautophagy, and unless otherwise specified, autophagy refers to macroautophagy. Autophagy begins with a phagosome formed by a double-layered membrane, wrapping some substances to be degraded to form an autophagosome, which fuses with a lysosome to form an autophagolysosome, which is eventually degraded (Mizushima and Komatsu, 2011). Mitophagy is a special type of selective autophagy that degrades damaged or aged mitochondria through receptor-mediated mechanisms to maintain mitochondrial homeostasis in cells (Boya et al., 2013; Ploumi et al., 2017). Although necessary for the survival of eukaryotic cells, autophagy is a double-edged sword that can also cause cell damage or even death (Chen et al., 2012). A growing body of evidence has confirmed that autophagy plays a pivotal role in bone metabolism, and abnormal autophagy can disrupt the balance of bone metabolism. Wear particles accelerate the PPO process by affecting autophagy (Ha et al., 2014; Camuzard et al., 2019).

In this regard, a large number of studies have focused on RCD in PPO. Zhang R et al. (Zhang et al., 2021) conducted a bibliometric study on the current PPO research based on VOSviewer, and found that “autophagy”, “bone-resorbing cells” as well as “proinflammatory cytokines” are research hotspots in this field. Due to the duality of autophagy and the complexity of its regulatory mechanism, there is no exact consensus on its mechanism of action on PPO. Inappropriate levels of autophagy may lead to negative effects. Collectively, this review article focuses on the role of autophagy in the development and progression of PPO, so as to provide novel ideas for reducing the incidence of PPO and AL.

## 2 Cell types involved in PPO

### 2.1 Osteocytes

Osteocytes are embedded in the bone matrix and account for more than 95% of all osteocytes in adult bones. They are terminally differentiated cells of the osteoblast lineage and are involved in bone growth, bone modeling, and bone remodeling (Bonewald, 2007; O'Brien et al., 2013). Sclerostin (encoded by SOST), a potent Wnt/β-catenin inhibitor, is a soluble glycoprotein secreted only by osteocytes that controls bone formation and absorption by binding to the co-receptor low-density lipoprotein receptor-related protein (LRP5/6). When osteocytes are mechanically stimulated, sclerostin production is blocked to activate osteoblasts to promote new bone formation (Lu et al., 2018). In addition, Osteoprotegerin (OPG) and receptor activator of nuclear factor kappa B ligand (RANKL) expressed by osteocytes are also directly involved in the regulation of bone resorption (Nakashima et al., 2011).

### 2.2 Osteoblasts and osteoclasts

As important cellular components of bone, osteoblasts and osteoclasts work together to maintain bone metabolic homeostasis. Osteoblasts are differentiated from bone marrow mesenchymal stem cells (BMSCs), which is the important source of osteocytes. Osteoclasts, the only cells responsible for bone resorption, are multinucleated giant cells derived from monocytes or macrophages (Madel et al., 2019). Therefore, the generation and activity enhancement of osteoclasts play a key role in the pathogenesis of PPO (Jacome-Galarza et al., 2019). Osteoblasts and chondrocytes originated from BMSCs are responsible for the construction of bone. On the contrary, their “enemies” are osteoclasts derived from hematopoietic stem cells, responsible for bone resorption and destruction (osteolysis). They jointly maintain the homeostasis of the bone matrix (Zaidi, 2007). Due to the close expression profile of osteoclasts, the RAW 264.7 cell line is often used by scholars in experiments to demonstrate the characteristics of osteoclasts (Todde et al., 2009).

RANKL and M-CSF are essential cytokines for the activation and maturation of osteoclast precursors (Zauli et al., 2004; Chakraborty et al., 2017). M-CSF upregulates the expression of RANK, the cognate receptor for RANKL (Arai et al., 1999; Novack and Mbalaviele, 2016). RANKL is a member of the TNF family. After binding to the specific receptor RANK, it activates the downstream nuclear factor-κB (NF-κB) and mitogen-activated protein kinase (MAPK) signal transduction pathways through recruiting the intracellular adaptor protein TNF receptor-associated factor (TRAF) (Arai et al., 1999; Feng, 2005). The mechanisms of the interaction between osteoblasts and osteoclasts are complex. OPG, also known as osteoclastogenesis inhibitory factor, secreted by osteoblasts or BMSCs, is a secreted glycoprotein that also belongs to the TNF receptor family. OPG is a decoy receptor for RANKL and inhibits osteoclast differentiation and osteolysis by blocking the interaction of RANKL with RANK by competing with RANK for RANKL binding (Kobayashi et al., 2009).

### 2.3 Fibroblasts

In PM, the highest proportion is fibroblasts, reaching more than 70%, however, much less attention has been paid to its role in PPO (Wei et al., 2005). When stimulated by wear particles, fibroblasts or fibroblast-like synoviocytes (FLSs) can not only secrete inflammatory factors, including RANKL, IL-6, IL-8, IL-1β and TNF-α, *etc.*, to promote the maturation of osteoclasts (Wei et al., 2005), but also can produce sclerostin (SOST), an antagonist of Wnt and BMP signaling, to inhibit the osteogenic capacity of osteoprogenitor cells, thereby exacerbating bone loss (Jagga et al., 2021). In addition, it was found that nano-Al_2_O_3_ particles reduced the expression of RANKL by inducing autophagy in fibroblasts, thereby alleviating PPO (Li et al., 2018). In-depth study of fibroblasts contributes to further understanding of the pathogenesis of PPO.

## 3 Autophagy

Autophagy has been extensively studied in osteolysis and PPO. Although most scholars believe that wear particles can induce an increase in the level of autophagy and accelerate osteolysis, some scholars hold the opposite opinion, arguing that autophagy has a protective effect, and enhancing autophagy can prevent PPO (Wang et al., 2017; Li et al., 2018). Autophagy is induced in a RANKL-dependent manner during osteoclastogenesis and also strongly influences the process of osteoclastic bone resorption (Zhang et al., 2019).

**FIGURE 1:**
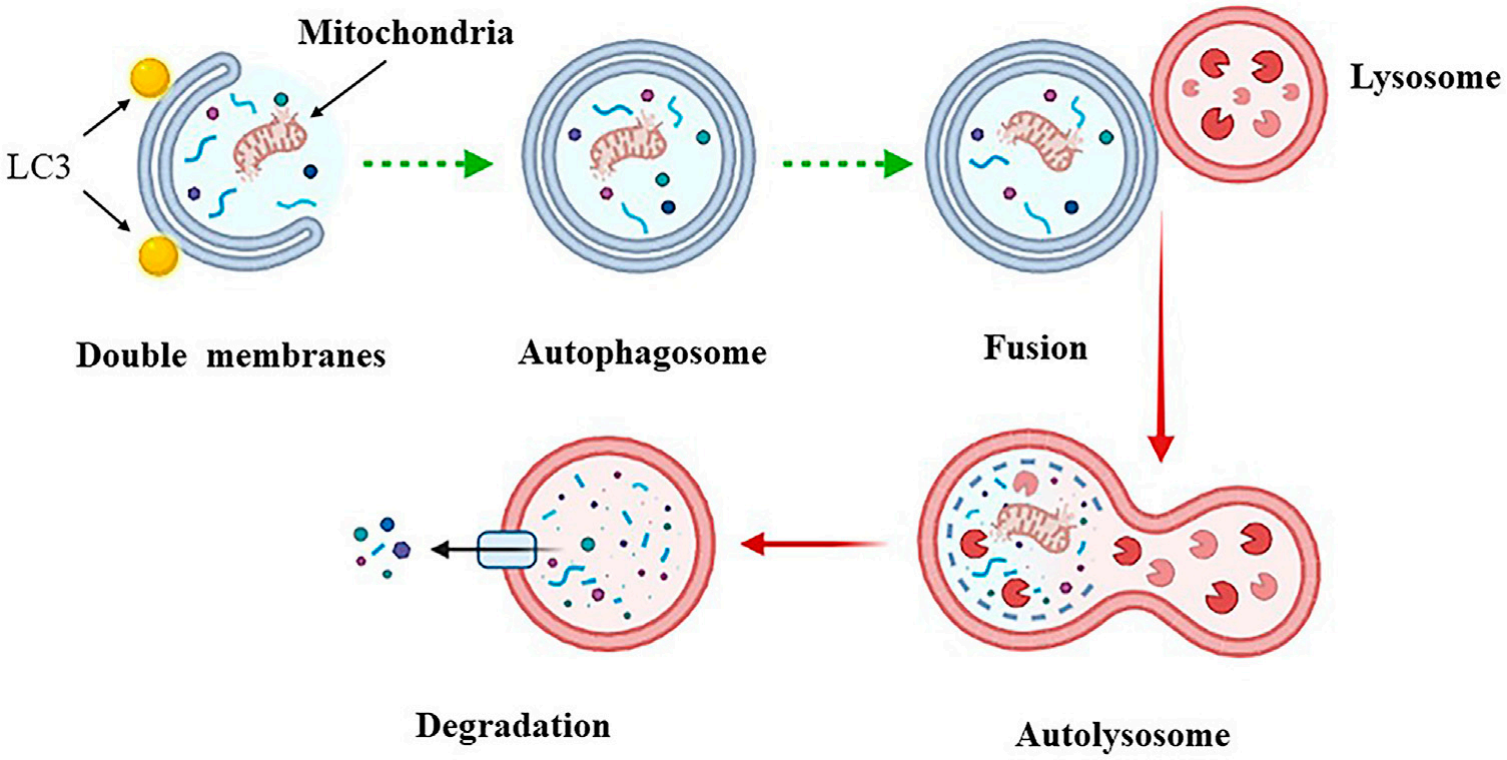
The process of autophagy.

The term “autophagy” is originated from the Greek meaning for “eating of self”. In the 1950s, Belgian scientist Christian de Duve used electron microscopy to observe the structure of autophagosomes, and in 1962, Ashford et al. observed autophagy in mouse liver cells after insulin treatment using transmission electron microscopy (ASHFORD and PORTER, 1962; Deter and De Duve, 1967). Autophagy is a highly conserved autocatabolic cellular process unique to eukaryotic cells. It degrades cytoplasmic misfolded or aggregated proteins, damaged organelles, long-lived proteins or exogenous substances through the lysosomal system to achieve the metabolic needs of the cells themselves, which is important to maintain the homeostasis of cells (Pohl and Dikic, 2019). The process of autophagy occurrence can be divided into four stages: Ⅰ. formation of septum membrane; Ⅱ. formation of autophagosome; Ⅲ. transportation and fusion of autophagosome; Ⅳ. lysis of autophagosome (Figure 1). Under normal physiological conditions, the body keeps a low level of autophagy to maintain cell metabolism and organelle renewal. When stimulated by the external environment (such as lack of oxygen, sugar, amino acid, and energy, *etc.*), it will activate intracellular autophagy, and then encapsulate the degraded material with double membranes to form autophagosomes, which are transported to lysosomes. Subsequently they combine to form autophagolysosomes, which are digested and degraded by a variety of enzymes and at the same time provide raw materials for the synthesis of new organelles to maintain cell survival (Rosenfeldt and Ryan, 2009; Rubinsztein et al., 2012). However, when autophagy is overactivated, resulting in mitochondrial dissolution and denaturation, chromatin fragmentation, and nuclear disintegration, it will cause cell death, which is called autophagy-dependent cell death (Rabinovich-Nikitin and Kirshenbaum, 2021).

ATG proteins transcribed and translated by autophagy related genes (*Atg*) are involved in the initiation, formation and eventual degradation of autophagosomes. *Atg3*, *Atg5*, *Atg7*, *Atg10*, *Atg12* and LC3 (microtubule-associated protein 1 light chain 3, MAP1-LC3) are involved in two ubiquitin-like protein processing and modification processes (Alonso et al., 2007). *Atg12* is localized on the bilayer isolation membrane of preautophagosomes and is associated with the formation of preautophagosomes. LC3, homologous to yeast Atg7/8, is localized to the autophagosome separation membrane (not bound to phosphatidylethanolamine [PE]), inner and outer membranes, and autophagolysosomal membranes (bound to PE) (Wu et al., 2006). After the LC3 precursor is formed, it is processed by the protease ATG4 into cytoplasmic soluble LC3-I (18 kDa), which can combine with PE on the surface of the autophagic vesicle to form the membrane-bound form LC3-II (16 kDa). LC3-II, a marker molecule of autophagosomes, is located in pre-autophagosomes and autophagosomes, and its content is proportional to the number of autophagosomes (Liu et al., 2017).

In mammals, autophagy is mainly divided into microautophagy, macroautophagy and chaperone-mediated autophagy (Parzych and Klionsky, 2014). The aforementioned autophagy refers to macroautophagy. Microautophagy and endosomal microautophagy refers to the process in which the cargoes are internalized directly by invaginations of lysosome and endosomal membranes (Mijaljica et al., 2011; Tekirdag and Cuervo, 2018). Molecular chaperone-mediated autophagy means that proteins are recognized by cytoplasmic partners one by one and bring them to the lysosomal surface for transmembrane translocation (Dice, 2007; Kaushik and Cuervo, 2018). Unless otherwise specified, the autophagy referred to in this article is macroautophagy.

Accumulating evidence suggests that autophagy is an important etiology of osteolysis. Autophagy can be elicited by wear particles in the many cell types involved in osteolysis, including osteoclasts, osteoblasts, osteocyte, fibroblasts and macrophages (Wang et al., 2020). Studies found that the level of autophagy increased during osteoblast differentiation. deletion of either *Atg5* or *Atg7* resulted in reduced osteoblast numbers in mice (Vrahnas et al., 2019). Interestingly, in an osteolysis model, Wang Z and his researchers demonstrated that apoptosis induced by CoCrMo metal particles exacerbated osteolysis by stimulating osteoblast autophagy (Wang et al., 2015a). The same research team also confirmed that TiAl_6_V_4_ particles induced autophagy in osteocytes mediated the downregulation of IFN-β, which in turn activated osteoclasts (Wang et al., 2017). In arthritis models, inhibition of autophagy limited macrophage differentiation to osteoclasts, thereby attenuating joint destruction (Shi et al., 2017). Nevertheless, nano-Al_2_O_3_-induced autophagy of fibroblasts limited osteoclast activation by reducing RANKL expression (Li et al., 2018). Therefore, modulation of autophagy on different types of cells in PM plays different roles in the treatment of PPO.

During the process of osteoblast differentiation, mitochondrial function and ATP content were significantly enhanced (Gao et al., 2018). Mitophagy was first proposed by Lemasters in 2005 (Lemasters, 2005). Autophagy is divided into selective autophagy and non-selective autophagy. Mitophagy belongs to the latter. PTEN-induced putative kinase 1 (PINK1), a serine/threonine (Ser/Thr) kinase, is required in the Parkin mediated mitophagy (Kawajiri et al., 2010; Narendra et al., 2010). Parkin is an E3-ubiquitin ligase selectively recruited to dysfunctional mitochondria by PINK1 *via* phosphorylation of Ser 65 in the UBL domain of Parkin (Narendra et al., 2008; Kondapalli et al., 2012). Similarly, mitophagy contributes to tissue homeostasis by reducing reactive oxygen species (ROS) and pro-inflammatory factors produced by damaged mitochondria. *In vitro*, 17β-estradiol enhances mitophagy in the mouse osteoblast cell line MC3T3-E1 to promote cell proliferation (Sun et al., 2018). However, mitophagy disturbances may also impair cellular energy metabolism and physiological functions (Goldman et al., 2010). One study unmasked that inhibition of autophagy is accompanied by endoplasmic reticulum and mitochondrial accumulation in Atg7-deleted osteoblasts, resulting in decreased bone mass and increased fractures in mice (Piemontese et al., 2016). Mitochondrial fusion proteins MFN1 (mitofusin1) and MFN2 are GTPases located on the outer mitochondrial membrane and play a key role in mitochondrial fusion. MFN2 can also promote mitophagy (Chen et al., 2003; Zhao et al., 2011). Ballard A et al. (Ballard et al., 2020) discovered that in osteoclast precursors with a double conditional knockout of MFN1 and MFN2, osteoclasts maturation was suppressed and bone mass was increased in female mice. In addition, MFN2-deficient female mice were resistant to Rankl-induced osteolysis and mitophagy was essential for osteoclast differentiation. The duality of autophagy remains controversy in the pathogenesis of osteolysis, but it is undeniable that the regulation of autophagy is a powerful weapon in the prevention and treatment of osteolysis.

## 4 Autophagy in osteolysis

### 4.1 Autophagy promotes osteolysis (negative effect)

Netrin-1 is an axon guidance protein involved in osteoclast activation and osteolysis (Purdue et al., 2007). L. Wang et al. (Wang et al., 2021) found that Ti particles activated autophagy in RAW 264.7 cells by increasing the expression of Netrin-1 and its receptor Unc5b, thereby promoting the expression of Atg5, Atg7, Atg12, Beclin-1 and LC3-II. Although the autophagy inhibitor 3-methyladenine (3-MA) reversed Ti particle-induced TRAP, RANKL and OPG expression and osteoclastogenesis, it did not affect Netrin-1. Netrin-1 promotes autophagy in RAW 264.7 *via* ERK phosphorylation, which subsequently induces osteoclastogenesis and increases in inflammatory factors such as IL-1β, IL-6, and TNF-α.

A certain level of transcription factor (NF)-κB is necessary to normal bone metabolism (Abu-Amer, 2013), however, under inflammatory conditions, high level of NF-κB causes osteoclast activation and bone destruction (Xing et al., 2005; Pasparakis, 2008). NF-κB is the principal mediator of RANK signaling, and its signaling pathway members include NF-κB1 (p50), NF-κB2 (P52), RelA (p65), IκB, IKKα, IKKβ, and NEMO (Franzoso et al., 1997; Boyce et al., 1999; Boyce et al., 2010). Normally, NF-κB exists as a dimer. When unstimulated, the nuclear localization sequence of NF-κB binds to inhibitory IκB proteins. Stimulatory signals, such as RANKL, cause IKKγ/NEMO and IKKβ, as well as other adaptor proteins, to assemble into IκB kinase (IKK), which phosphorylates the downstream substrate IκB (Franzoso et al., 1997; Boyce et al., 2005; Boyce et al., 2010), and lead to the release of the p65/p50 NF-κB subunits into the nucleus to exert their functions (Gilmore, 2006). Phosphorylated IκB are degraded through the proteasomal pathway. The ubiquitin-proteasome system plays a crucial role in regulating NF-κB activity (Skaug et al., 2009).

Stimulation of bone marrow derived macrophages (BMDMs) by RANKL is multifaceted. Apart from NF-κB, RANKL stimulation identically activates the MAPK signalling pathway including ERK, JNK and p38. The activation of the above two signaling pathways requires TRAF2, TRAF5 and TRAF6, especially TRAF6 (Feng, 2005). TRAF6 deficiency facilitates osteosclerosis in mice (Lomaga et al., 1999). RANKL activates RANK in a trimeric symmetric complex with TRAF6, a RING-domain E3 ubiquitin ligase (Yu et al., 2018), together with NFATc1, are essential for osteoclast maturation and differentiation (Kim et al., 2009). In addition, TRAF6 recruitment after RANKL stimulation resulted in further activation of NF-κB, MAPK (JNK, ERK and p38) and Akt (Kim et al., 2013). In a recent study, it was found that in the RANKL-RANK signaling pathway, TRAF6 binds and induces the ubiquitination of Beclin- 1, which promotes autophagy and induces osteoclast differentiation (Chu et al., 2020). One research conducted by B. Chu et al. (Chu et al., 2020) indicated that application of Nepetin, A flavonoid with anti-inflammatory activity, not only inhibited RANKL-induced activation of NF-κB and MAPK, but also impeded TEAF6-dependent Beclin-1 ubiquitination and autophagy induction, which are necessary for osteoclast differentiation. Nepetin prevented IκBα degradation as well as p65 nuclear localization by inhibiting IKK activation, thereby reducing the transcriptional activity of NF-κB and the induction of c-Fos and NFATc1. Intriguingly, the inducer of autophagy, rapamycin, counteracted the inhibitory effect of Nepetin on autophagy and accelerated osteoclast activation and bone resorption. This study further elucidated the potential therapeutic application of autophagy inhibition against osteoclast-dependent osteolysis.

Bortezomib (BTZ), a reversible proteasome inhibitor, is recognized to hinder nuclear transport of NF-κB by impeding the chymotryptic activity of polyubiquitinated protein degradation in 26S proteasome, thereby inhibiting NF-κB (Deng et al., 2017a; Grosset et al., 2019). Zhang Z and his research team (Zhang et al., 2020a) found that Ti particles caused MG-63 cells, a type of osteosarcoma-derived cells that resemble poorly differentiated osteoblasts, to activate the NF-κB signaling pathway and increase inflammatory mediators, such as IL-1β, IL-6, TNF-α, release along with activation of the autophagy marker LC3. The use of nano-aluminum particles and BTZ, a proteasome inhibitor, co-cultured with MG-63 blocked the degradation of IκBα induced by Ti particles and inhibited the activation of NF-κB. Intriguingly, Nano-Alumina (Al) inhibited autophagy and prevented apoptosis and osteolysis. Using nano-Al to improve the pro-inflammatory properties of Ti particles, combined with inflammatory factor inhibitors to regulate autophagy may be an effective way to alleviate osteolysis.

Although osteocytes are the most numerous cells in the bone, the relationship between osteocytes and osteoclasts in periprosthetic osteolysis studies has not been well studied. Previous studies have demonstrated that ultra-high molecular weight polyethylene (UHMWPE) particles stimulate osteocytes to secrete prostaglandin E2 to activate osteoclasts (Lohmann et al., 2002). IFN-β, a pleiotropic cytokine in cellular immunity, is also responsible for osteoclast differentiation and bone resorption (Abraham et al., 2009; Xiong et al., 2016). IFN-β can hinder RANKL-induced osteoclast activation through multiple signaling pathways, such as RANKL-c-fos-NFATc1 (Zhao et al., 2014). Wang Z et al. (Wang et al., 2017) reported that TiAl_6_V_4_ alloy particles enhanced the autophagy level of osteocytes and decreased the expression of IFN-β, which inhibited the differentiation of BMDMs into osteoclasts. Negatively modulation of autophagy with Atg5 siRNA blocked these effects. Another study by the same researching group unmasked that TiAl_6_V_4_ induced autophagy in macrophages and phosphorylates p38, thereby upregulating the expression of TNF-a and accelerating osteolysis. The autophagy inhibitor 3-MA or p38 inhibitor SB202190 hindered the above process (Liu et al., 2016). Intriguingly, another research by the same study group confirmed that CoCrMo particles promoted osteoblast apoptosis in osteoblastic cell line MC3T3-E1 by facilitating autophagy through ERN1-MAPK8 pathway. Similarly, Atg5 siRNA and 3-MA attenuated CoCrMo particles-induced osteoblast apoptosis and osteolysis by the impeding of the autophagy (Wang et al., 2015a). Another experiment using the MC3T3-E1 cell line implied that hyperoside pretreatment protects against Ti particle-induced damage by reducing osteoblast autophagy and apoptosis through the tumor necrosis factor ligand superfamily member 12 (TWEAK)-p38 pathway (Zhang and Zhang, 2019).

Earlier studies indicated that titanium particles induced increased expression of CD147, a transmembrane glycoprotein belonging to the immunoglobulin superfamily, in KG-1a cells, which activated autophagy, thereby increasing soluble RANKL levels and promoting osteoclast occurrence. These effects were reversed when siRNA-CD147 transfection or autophagy inhibitor chloroquine were performed (Su et al., 2018). Consequently, blocking autophagy may represent a potential therapeutic approach for treating particle-induced PPO.

### 4.2 Autophagy inhibits osteolysis (positive effect)

As important players in the inflammatory response, fibroblasts play an important role in chemokine synthesis and inflammation regulation (Smith et al., 1997; Flavell et al., 2008). Studies have confirmed that wear particles induce an increase in the RANKL/OPG ratio in fibroblasts (Wang et al., 2015b). When activated, cytokines secreted by fibroblasts induce the recruitment and differentiation of monocytes, a process involved in wear particle-mediated osteolysis (Goodman and Ma, 2010; Filer, 2013). Fibroblasts occupy a large amount in the pseudo-synovia around the joint after joint replacement, and their secretion of RANKL promotes the activation of osteoclasts under the stimulation of titanium particles (Wei et al., 2005). As touched upon earlier, Al reduced the inflammatory response of cells induced by Ti particles (Zhang et al., 2020a). Strikingly, unlike aluminum that inhibited autophagy, some scholars proposed that aluminum promoted autophagy, although the former was detected in osteoblast-like MG-63 cells (Zhang et al., 2020a) and the latter was in FLSs (Li et al., 2018). D Li and others (Li et al., 2018) isolated FLSs from the synovium of clinical revision arthroplasty patients and stimulated them with Al_2_O_3_, CoCr, and UHMWPE particles, respectively. showed lower levels of RANKL, autophagy, and inflammation. The results demonstrated that the increased autophagy intensity of FLSs was accompanied by lower RANKL expression after Al_2_O_3_ stimulation. Lentiviral interference or enhancement of Beclin-1 further confirmed that autophagy levels were inversely proportional to RANKL expression and the number of osteoclasts. Afterwards, in a rat model of femoral head replacement, Al_2_O_3_ reduced FLSs secretion of RANKL and osteoclast activation by enhancing autophagy. Although the regulation of autophagy by aluminum is controversial, these conclusions provide further consideration for the selection of joint prosthesis materials.

Wear particle-mediated monocyte recruitment and inflammatory responses are important pathogenesis of osteolysis. In an earlier study in an interface membrane specimen from clinical joint prosthesis revision, titanium particle stimulation inhibited autophagy in fibroblasts and resulted in ADAM metallopeptidase domain 10 (ADAM10), including the precursor form p-ADAM10 and its mature form m-ADAM10 increase. Subsequent secretion of C-X3-C motif chemokine ligand 1 (CX3CL1), a unique chemokine, increased and promoted the migration of the human myeloid leukemia mononuclear (THP-1) cells. The autophagy activator rapamycin reversed the effects of titanium particles (Wu et al., 2020). Accordingly, blocking monocyte recruitment and the inflammatory response it induces is also an important strategy to prevent osteolysis.

**FIGURE 2:**
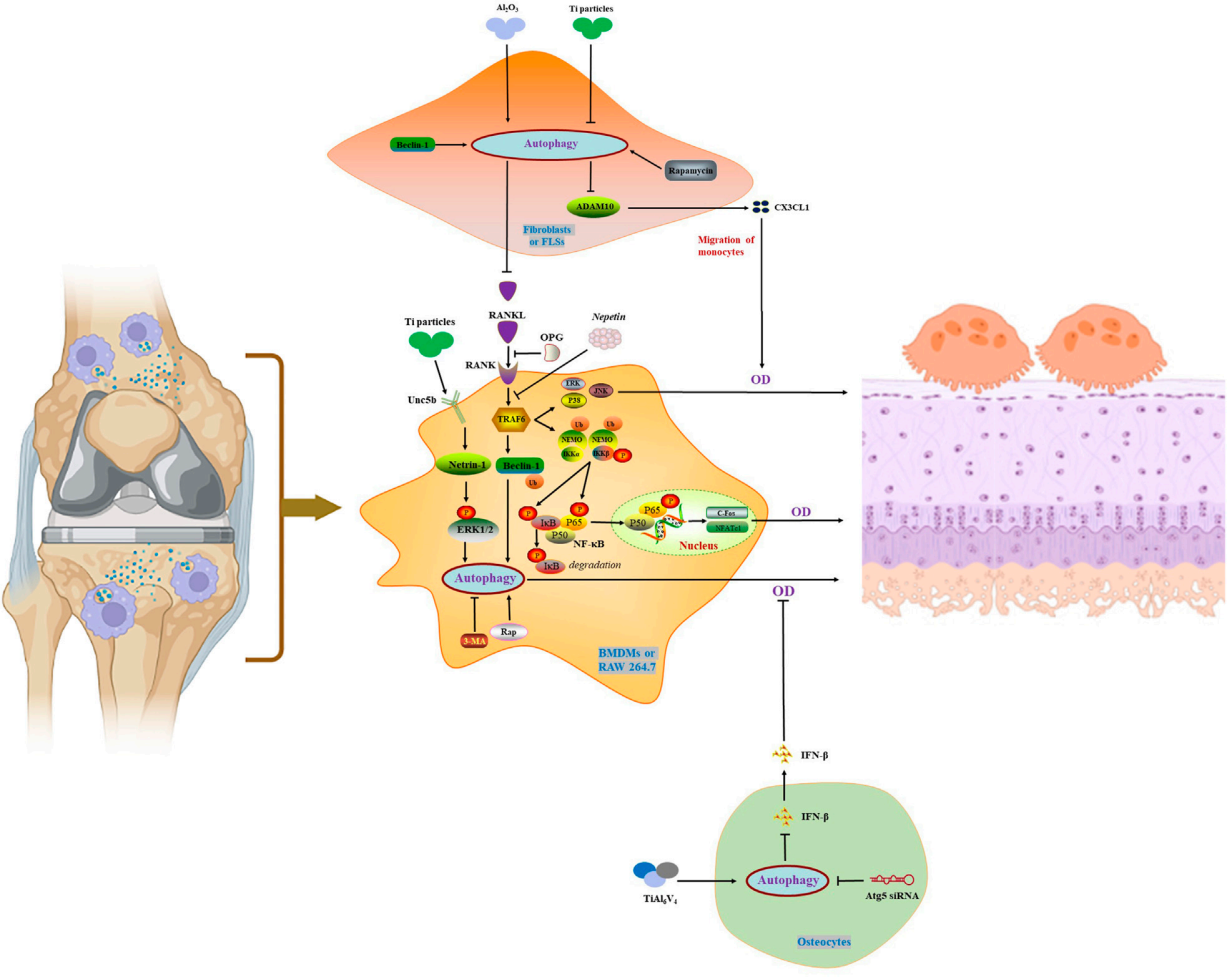
Autophagy in the activation and maturation of osteoclasts.

As touched upon above, NEMO, a scaffold protein lacking enzymatic activity, is a key molecule in the NF-κB signaling pathway. Adapala N et al. (Adapala et al., 2020) identified lysine K)270 as a target regulating RANKL signaling, and NEMO^K270A^ mutation hinders autophagy in BMDMs and exacerbates rankl-induced osteoclastogenesis. Additionally, the molecular mechanisms and the treatment strategies mentioned above to regulate autophagy for the treatment of PPO and AL were summarized in Figure 2 and Table 1.

**TABLE 1:** Experimental study of autophagy in PPO.

Intervention target/agents	Animal	Cell type	Animal model	*In vitro* cell model	Hallmark of autophagy	Molecular mechanism	Ref
Netrin‐1	Mice	RAW 264.7	An air pouch model	Stimulated by Tiparticles	Atg5; Atg7;Atg12;LC3 Beclin-1	Ti particles induce osteoclastactivation by activating Netrin-1 andits Unc5b receptor to phosphorylateERK1/2 and facilitate autophagy	Kawajiri et al., (2010)
Nepetin	C57BL/6 mice	BMDMs andRAW 264.7	Calvarial osteolysisinduced by Tiparticles	Stimulated byRANKL	Atg5; Atg12;Beclin-1; LC3	Nepetin inhibits RANKL-RANK-induced TRAF6 recruitment andimpedes Benlin-1 ubiquitination andautophagy	Boyce et al., (1999)
Al particles and BTZ	C57BL/6 mice	Humanosteoblast-likeMG-63 cells	Calvarial osteolysisinduced by Tiparticles	Stimulated by Tiparticles	LC3	Aluminum nanoparticles and theproteasome inhibitor BTZ inhibitautophagy and NF-κB activation toprevent apoptosis and osteolysis	Boyce et al., (2005)
siAtg5	C57BL/6 mice	Osteocytic cellline MLO-Y4 andBMDMs	$\text{Calvarial osteolysis}{\text{induced by TiAl}}_{6}{\text{V}}_{4}\text{particles}$	$\text{MLO-Y4:}\text{stimulated by}{\text{TiAl}}_{6}{\text{V}}_{4}\text{particles}$	LC3	${\text{TiAl}}_{6}{\text{V}}_{4}\text{ particles enhanced osteocyte}\text{autophagy to reduce IFN-β expression}\text{and increase osteoclastogenesis.}\text{Atg5 siRNA inhibits autophagy and}\text{the differentiation of BMDMs into}\text{osteoclasts}$	Kang et al., (2017)
				BMDMs:stimulated byM-CSF andRANKL			
3-MA	C57BL/6 mice	Peritonealmacrophages	$\text{Calvarial osteolysis}{\text{induced by TiAl}}_{6}{\text{V}}_{4}\text{particles}$	Not mentioned	LC3	${\text{TiAl}}_{6}{\text{V}}_{4}\text{ upregulates TNF-a}\text{expression and accelerates osteolysis}\text{by inducing autophagy and}\text{phosphorylating p38}$	Kim et al., (2009)
3-MA; siAtg5	C57BL/6 mice	Osteoblastic cellline MC3T3-E1	Calvarial osteolysisinduced byCoCrMo particles	Stimulated byCoCrMoparticles	LC3	CoCrMo particles promote osteoblastapoptosis by enhancing autophagythrough ERN1-MAPK8 pathway	Wu et al., (2006)
Chloroquine	Not mentioned	The cell line KG-1a (macrophage)	Not established	Stimulated by Tiparticles	Beclin-1	Titanium particles activate autophagyby inducing increased expression ofCD147, thereby increasing RANKLlevels and promotingosteoclastogenesis	Chu et al., (2020)
${\text{Nano-sized Al}}_{2}{\text{O}}_{3}\text{particle}$	Human(ObtainingFLSs);SD rats	FLSs from clinical	Femoral headreplacement modelstimulated by Alparticles	Stimulated by Alparticles	Atg5; Beclin-1;LC3; p62	${\text{Al}}_{2}{\text{O}}_{3}\text{ reduces RANKL secretion and}\text{inhibits osteoclast activation by}\text{enhancing autophagy}$	Zauli et al., (2004)
Rapamycin	Human(Obtainingfibroblasts)	Fibroblasts fromclinical	Not established	Stimulated by Tiparticles	LC3	Titanium particles inhibitesautophagy in fibroblasts, resulting inincreased ADAM10 expression, whichsubsequently promotesCX3CL1 release and chemotacticmigration of THP-1. Rapamycinreverses this effect	Zhao et al., (2014)

Red: Autophagy promotes PPO.

Blue: Autophagy relieves PPO.

Precision therapy, such as targeted drug delivery, is a hot spot in modern medical research (Li et al., 2021). A recent experiment released reactive oxygen species (ROS) scavenger and autophagy activator rapamycin through a drug-loaded system on a nanoplatform to rescue chondrocyte degeneration after IL-1β stimulation for the treatment of osteoarthritis (Xue et al., 2021). Thus, At the same time of primary joint replacement, local implantation of drug release system to alleviate the inflammatory response caused by later prosthesis wear is still a therapeutic strategy worthy of further study.

## 5 Disscussion

Although joint replacement has greatly improved the quality of life of patients with severe end-stage joint disease, aseptic osteolysis and loosening of the prosthesis caused by wear particles of the prosthesis limit the service life of the artificial joint (Varnum, 2017). Various cell types, including osteoblasts, osteoclasts, macrophages, fibroblasts, and lymphocytes, are involved in the pathological process of PPO, and the direct factor is the imbalance between osteoblasts and osteoclasts. RANKL/RANK/OGP axis is a typical way for RANKL to participate in bone remodeling. The role of autophagy has aways been controversial, and there is no clear conclusion so far. It is undeniable that wear particles can induce the occurrence of autophagy in PM. Autophagy induces macrophages and osteoblasts to secrete proinflammatory cytokines, such as TNF-α, IL-6 and IL-8 or high mobility group box 1, which exacerbate the progression of PPO (Camuzard et al., 2019). However, autophagy can also inhibit the release of RANKL from fibroblasts or reduce the expression of IFN-β by osteoblasts, thereby hindering the activation of osteoclasts (Li et al., 2018; Wu et al., 2020). In addition, 17β-estradiol alleviates osteoporosis by enhancing osteoblast autophagy through G protein-coupled receptor 30-extracellular regulated protein kinase 1/2 (ERK1/2) signaling pathway, which also exhibits positive effect of autophagy (Sun et al., 2018). During the progression of PPO, complex regulatory mechanisms may exist between cells in the PM, including crosstalk in autophagy regulation. It is worth noting that different autophagy intensities and durations may have opposite effects. The research on autophagy in PPO mainly focuses on macroautophagy, chaperone-mediated autophagy and microautophagy have not yet been studied, and mitophagy is also a research gap, which warrants deeper examination. NF-κB activation is critical for the recruitment and maturation of macrophages, as well as the production of pro-inflammatory cytokines and chemokines (Lin et al., 2014). The regulation of autophagy on the NF-κB pathway in PPO deserves further study. Autophagy has a dual role in PPO, how to rationally regulate autophagy to prevent PPO remains to be fully elucidated.

In contrast, the pathogenesis of selective autophagy in PPO has been less studied. As the site of cellular energy conversion, the integrity and quantity of mitochondria play an active role in the physiological functions of cells. When mitochondrial dysfunction occurs, it is manifested by increased release of ROS and inflammatory factors, mitochondrial depolarization and enhanced mitophagy (Guo et al., 2011; Hui et al., 2016; Collins et al., 2018). Regulation of mitochondrial number and function through mitophagy is important for maintaining the biological activity of osteoblasts and osteoclasts. Another form of selective autophagy, ferroautophagy, is mediated by nuclear receptor coactivator 4 (NCOA4), enabling ferritin, mainly ferritin heavy chain 1, to degraded by autophagosomes. Eventually ferritin-bound iron is released as free iron. Excessive activation of ferroautophagy may induce intracellular iron overload and lead to cellular damage (Bauckman and Mysorekar, 2016; Nai et al., 2021). X Qin et al. revealed that ferroautophagy was necessary for zinc oxide nanoparticles-induced ferroptosis in vascular endothelial cells (Qin et al., 2021). Ferroautophagy is involved in processes including tumor development and erythropoiesis (Zhang et al., 2020b; Nai et al., 2021), however, its effect on PPO deserves further study.

Strikingly, most studies hinder the progress of PPO by enhancing or inhibiting autophagy, which further verifies the duality of autophagy. Nevertheless, it is difficult to achieve therapeutic effects by simply regulating autophagy because of complex cell differences as well as differences in time and space. Similarly, autophagy also has both a “dark” and a “bright” side in tumor therapy. Autophagy and mitophagy are thought to contribute to drug resistance and survival of cancer cells. Mitophagy reduces ROS formation by degrading damaged mitochondria, and anticancer drugs combined with autophagy inhibition have been validated in clinical trials of various human cancers. However, mitophagy inhibition also promotes cancer cell metastasis (Erkan et al., 2005; Ishaq et al., 2020). As a result, simply enhancing or inhibiting autophagy may not achieve satisfactory therapeutic effects. Interfering with autophagy at different times during the PPO process may have opposite effects. Even if autophagy is intervened at the same time, autophagy in different cells may play different roles in PM. The precision of time and space, the appropriate strength of autophagy, and the precise targeting of cellularity will be an important aspect of future research, especially the exact intervention of autophagy in a specific cell.

There is an urgent need to find safe and effective therapeutic drugs to relieve PPO in clinical practice. Anti-rankl antibodies (Denosumab), parathyroid hormones (Teriparatide and Abaloparatide), bisphosphonates (Fosamax) and selective estrogen receptor modulators (Raloxifene) have shown promising anti-osteolytic effects by inhibiting osteoclast activity. Sclerostin modulators are also actively entering clinical applications. SOST modulators (Romosozumab and Blosozumab) are also actively entering clinical applications. Nevertheless, long-term use of these drugs may bring serious adverse reactions, such as cardiovascular accidents, liver and kidney toxicity, bone necrosis, and malignant tumors, which limit their clinical use (Herrington et al., 2000; Berruti et al., 2006; Cao et al., 2014; Hayden et al., 2014). In terms of autophagy regulation, reducing RANKL production by modulating autophagy in fibroblasts is also a potential therapeutic strategy. By developing new drugs targeting autophagy of different cells in PM, as well as changing the time and dose of administration, in order to more accurately control the process of PPO development. Improvements in prosthetic design and surgical modalities are additional potential approaches. Interestingly, compared with titanium particles, ceramic particles had less effect on the release of inflammatory factors and stimulation of macrophages (Zhang et al., 2011; Ding et al., 2012; Jeffers and Walter, 2012). Al_2_O_3_ can also inhibit osteoclast maturation by enhancing autophagy (Li et al., 2018). Therefore, optimizing the prosthetic material is also crucial to reduce the incidence of PPO.

Collectively, we review the critical roles of autophagy, mitophagy and ferroautophagy in the pathophysiology of PPO. The occurrence of PPO is the result of various intercellular interactions. Autophagy has two sides, and how to adjust the time and intensity of autophagy to reduce the occurrence of osteolysis remains challenging. Although several studies support the important role of autophagy in PPO, there are still many unanswered questions about the regulation of autophagy in timing, intensity and target cells. Understanding the molecular mechanism of autophagy in PPO will provides insight for AL therapeutics.
