# Supplementary material for: The dual role of autophagy in periprosthetic osteolysis
Source: Front Cell Dev Biol. 2023 Mar 24;11:1123753. doi: 10.3389/fcell.2023.1123753 (PMC10080036; doi:10.3389/fcell.2023.1123753)
Supplement: Supplementary file 1 [file Table1.DOCX]

Table.1 Experimental study of autophagy in PPO

| Intervention target/agents | Animal | Cell type | Animal model | In vitro cell model | Hallmark of autophagy | Molecular mechanism | Ref |
| --- | --- | --- | --- | --- | --- | --- | --- |
| Netrin‐1 | Mice | RAW 264.7 | An air pouch model | Stimulated by Ti particles | Atg5; Atg7; Atg12; LC3 Beclin-1 | Ti particles induce osteoclast activation by activating Netrin-1 and its Unc5b receptor to phosphorylate ERK1/2 and facilitate autophagy. | 82 |
| Nepetin | C57BL/6 mice | BMDMs and RAW 264.7 | Calvarial osteolysis induced by Ti particles | Stimulated by RANKL | Atg5; Atg12; Beclin-1; LC3 | Nepetin inhibits RANKL-RANK-induced TRAF6 recruitment and impedes Benlin-1 ubiquitination and autophagy. | 96 |
| Al particles and BTZ | C57BL/6 mice | Human osteoblast-like MG-63 cells | Calvarial osteolysis induced by Ti particles | Stimulated by Ti particles | LC3 | Aluminum nanoparticles and the proteasome inhibitor BTZ inhibit autophagy and NF-κB activation to prevent apoptosis and osteolysis. | 98 |
| siAtg5 | C57BL/6 mice | Osteocytic cell line MLO-Y4 and BMDMs | Calvarial osteolysis induced by TiAl_6_V_4_ particles | MLO-Y4: stimulated by TiAl_6_V_4_ particles;  BMDMs: stimulated by M-CSF and RANKL | LC3 | TiAl_6_V_4_ particles enhanced osteocyte autophagy to reduce IFN-β expression and increase osteoclastogenesis. Atg5 siRNA inhibits autophagy and the differentiation of BMDMs into osteoclasts. | 16 |
| 3-MA | C57BL/6 mice | Peritoneal macrophages | Calvarial osteolysis induced by TiAl_6_V_4_ particles | *Not mentioned* | LC3 | TiAl_6_V_4_ upregulates TNF-a expression and accelerates osteolysis by inducing autophagy and phosphorylating p38. | 103 |
| 3-MA; siAtg5 | C57BL/6 mice | Osteoblastic cell line MC3T3-E1 | Calvarial osteolysis induced by CoCrMo particles | Stimulated by CoCrMo particles | LC3 | CoCrMo particles promote osteoblast apoptosis by enhancing autophagy through ERN1-MAPK8 pathway. | 68 |
| Chloroquine | *Not mentioned* | The cell line KG-1a (macrophage) | *Not established* | Stimulated by Ti particles | Beclin-1 | Titanium particles activate autophagy by inducing increased expression of CD147, thereby increasing RANKL levels and promoting osteoclastogenesis. | 105 |
| Nano-sized Al_2_O_3_ particle | Human (Obtaining FLSs); SD rats | FLSs from clinical | Femoral head replacement model stimulated by Al particles | Stimulated by Al particles | Atg5; Beclin-1; LC3; p62 | Al_2_O_3_ reduces RANKL secretion and inhibits osteoclast activation by enhancing autophagy. | 52 |
| Rapamycin | Human (Obtaining fibroblasts) | Fibroblasts from clinical | *Not established* | Stimulated by Ti particles | LC3 | Titanium particles inhibites autophagy in fibroblasts, resulting in increased ADAM10 expression, which subsequently promotes CX3CL1 release and chemotactic migration of THP-1. Rapamycin reverses this effect. | 111 |

**Red: Autophagy promotes PPO**

**Blue: Autophagy relieves PPO**

**Abbreviations （in alphabetical order）:**

Al: alumina

BMDMs: bone marrow derived macrophages

BTZ: bortezomib

FLSs: fibroblast-like synoviocytes

Ti: titanium
