# Supplementary material for: The dual role of autophagy in periprosthetic osteolysis
Source: Front Cell Dev Biol. 2023 Mar 24;11:1123753. doi: 10.3389/fcell.2023.1123753 (PMC10080036; doi:10.3389/fcell.2023.1123753)
Supplement: Supplementary file 2 [file Table2.DOCX]

**Fig 1 The process of autophagy**

**Fig 2 Autophagy in the activation and maturation of osteoclasts**

***Negative effects of autophagy***

Ti particles phosphorylate and activate ERK1/2 by increasing the expression of Netrin-1 and its receptor Unc5b, which subsequently induces autophagy and promotes osteoclastogenesis and inflammatory factors (IL-1β, IL-6, and TNF-α) release. The above process can be counteracted by the autophagy inhibitor 3-MA.

Nepetin inhibits RANKL-RANK-induced recruitment of TRAF6, on the one hand, inhibits the activation of MAPK (JNK, ERK and p38) and NF-κB, on the other hand, hinders Benlin-1 ubiquitination and subsequent induction of autophagy, preventing osteoclasts maturation. The autophagy activator rapamycin can counteract the inhibition of autophagy by Nepetin.

***Positive effects of autophagy***

In FLSs, Al_2_O_3_ reduced the secretion of RANKL by enhancing autophagy and inhibited the activation of osteoclasts.

In fibroblasts, Ti particles negatively regulated autophagy and relieved its inhibition of ADAM10, which subsequently promoted CX3CL1 release and monocytes migration. Rapamycin reverses this effect by enhancing autophagy.

In osteocytes, TiAl_6_V_4_ particles enhanced the autophagy of osteocytes and decreased the expression of IFN-β, which inhibits the differentiation of BMDMs into osteoclasts.

**Abbreviations（in alphabetical order）:**

3-MA: 3-methyladenine

BMDMs: bone marrow‑derived macrophages

FLSs: fibroblast-like synoviocytes

IFN: interferon

OD: osteoclast differentiation

OPG: Osteoprotegerin

P: phosphorylation

Rap: rapamycin

Ti: titanium

Ub: ubiquitination
